# An AlphaFold Structure Analysis of COQ2 as Key a Component of the Coenzyme Q Synthesis Complex

**DOI:** 10.3390/antiox13040496

**Published:** 2024-04-21

**Authors:** María de los Ángeles Vargas-Pérez, Damien Paul Devos, Guillermo López-Lluch

**Affiliations:** 1 Departamento de Fisiología, Anatomía y Biología Celular, Centro Andaluz de Biología del Desarrollo (CABD), CSIC-UPO-JA, Universidad Pablo de Olavide, Centro de Investigación Biomédica en Red de Enfermedades Raras (CIBERER), Instituto de Salud Carlos III, Carretera de Utrera km1, 41013 Seville, Spain; mavarper@gmail.com; 2 Centro Andaluz de Biología del Desarrollo (CABD), CSIC-UPO-JA, Universidad Pablo de Olavide, Carretera de Utrera km1, 41013 Seville, Spain; dpdev@upo.es

**Keywords:** coenzyme Q, ubiquinol, mitochondria, rare disease, antioxidant

## Abstract

Coenzyme Q (CoQ) is a lipidic compound that is widely distributed in nature, with crucial functions in metabolism, protection against oxidative damage and ferroptosis and other processes. CoQ biosynthesis is a conserved and complex pathway involving several proteins. COQ2 is a member of the UbiA family of transmembrane prenyltransferases that catalyzes the condensation of the head and tail precursors of CoQ, which is a key step in the process, because its product is the first intermediate that will be modified in the head by the next components of the synthesis process. Mutations in this protein have been linked to primary CoQ deficiency in humans, a rare disease predominantly affecting organs with a high energy demand. The reaction catalyzed by COQ2 and its mechanism are still unknown. Here, we aimed at clarifying the COQ2 reaction by exploring possible substrate binding sites using a strategy based on homology, comprising the identification of available ligand-bound homologs with solved structures in the Protein Data Bank (PDB) and their subsequent structural superposition in the AlphaFold predicted model for COQ2. The results highlight some residues located on the central cavity or the matrix loops that may be involved in substrate interaction, some of which are mutated in primary CoQ deficiency patients. Furthermore, we analyze the structural modifications introduced by the pathogenic mutations found in humans. These findings shed new light on the understanding of COQ2’s function and, thus, CoQ’s biosynthesis and the pathogenicity of primary CoQ deficiency.

## 1. Introduction

Coenzyme Q (CoQ, ubiquinone) is a lipidic molecule that is present in the three domains of life. From a biochemical point of view, it is formed by a highly hydrophobic polyisoprenoid tail with a variable number of isoprene units depending on the species (10 in humans, thus called CoQ_10_), which are attached to a benzoquinone head with redox properties [1]. CoQ is of paramount importance because of its universal role in the respiratory chain as an electron carrier. However, additional functions have been proposed [1,2,3,4]. In bacteria, it has been associated with other processes such as the response to oxidative stress, formation of disulfide bonds and regulation of gene expression [5,6], whereas in eukaryotes, a plethora of both mitochondrial and extramitochondrial functions have been identified.

For instance, CoQ accepts electrons from dehydrogenases enzymes located in the mitochondria; hence, it is involved in multiple metabolic pathways, including biosynthesis of pyrimidines, sulfide detoxification and oxidation of fatty acids and proline and branched chain amino acids [7]. It also regulates the mitochondrial permeability transition pore (mPTP) and uncoupling proteins (UCPs) [2]. In all cell membranes, including mitochondria and plasma lipoproteins, it functions as an antioxidant, protecting against lipid peroxidation and reducing other antioxidants, such as vitamin E and ascorbate [8,9]. Additionally, CoQ promotes membrane stability and protects against ferroptosis [10,11].

CoQ biosynthesis is a highly conserved process that takes place in the inner mitochondrial membrane in eukaryotes and the cytosol in prokaryotes [12]. Despite the early discovery of CoQ in 1957 [13,14], there is still a limited understanding of its biosynthesis process, which includes several transmembrane and peripheral membrane proteins, hydrophobic intermediates, unidentified components and, at least in yeasts and mammals, the existence of a biosynthetic complex known as the CoQ-synthome or complex Q. The proteins involved in CoQ biosynthesis are generally known as Ubi proteins in prokaryotes and COQ proteins in eukaryotes [1,3,15]. Thirteen proteins encoded in the nucleus genome take part in this pathway in humans: COQ2, COQ3, COQ4, COQ5, COQ6, COQ7, COQ8A (ADCK3), COQ8B (ADCK4), COQ9, COQ10A, COQ10B, PDSS1 (DPS1) and PDSS2 (DLP1) [4]. Mutations in some of these proteins (COQ2, COQ4, COQ6, COQ7, COQ8A, COQ8B, PDSS1 and PDSS2) have been related to primary CoQ deficiency in humans, which encompasses rare autosomal recessive diseases characterized by a wide heterogeneity of symptoms, severity and age of onset, mainly affecting organs with high energy needs, such as the brain, inner ear, muscles, heart and kidneys [1,3].

COQ2 protein or 4-hydroxybenzoate polyprenyltransferase (EC 2.5.1.39) is a transmembrane protein inserted in the inner mitochondrial membrane that participates in the second step in CoQ synthesis, which is the condensation of the benzoquinone head and the polyisoprenoid tail [1,3,16,17]. Finally, the head undergoes a series of modifications: three hydroxylations catalyzed in humans by COQ6 (C5 hydroxylation), COQ7 (C6 hydroxylation) and COQ4 (C1 hydroxylation); three methylations performed by COQ3 (C5 and C6 O-methylations) and COQ5 (C2 methylation); and a decarboxylation that has recently been associated with COQ4 (C1 decarboxylation). All these enzymes are peripheral membrane proteins located in the matrix side of the inner mitochondrial membrane [1,3,18].

In humans, the CoQ-synthome is thought to be constituted by the enzymes involved in head modification (COQ3, COQ4, COQ5, COQ6 and COQ7), together with other COQ proteins (COQ8A, COQ8B and COQ9), lipids (CoQ intermediates and phospholipids), cofactors and metal ions. This organization permits CoQ intermediates to be channeled from one enzyme to the next, reducing the risk of leakage. However, it is still unknown how the CoQ-synthome, composed mainly of peripherally associated proteins, comes into contact with the membrane-embedded intermediates, which are tightly located on the inner mitochondrial membrane [3]. COQ2 could function as an anchor site for the CoQ-synthome, as proposed in yeast by Tran and Clarke (2007) [19], making the substrates accessible to the head-modifying enzymes and, thus, solving potential transport problems associated with the hydrophobic nature of CoQ intermediates [3]. Nevertheless, no experimental 3D structure of human COQ2 is available to date, which hinders progress in understanding the function of this protein. Structural data on COQ2 and determining the position and structural effect of pathological mutations would, therefore, provide a valuable insight into CoQ’s biosynthesis and the pathogenicity of primary CoQ deficiency.

Taking advantage of the AlphaFold Protein Structure Database [20], our aim was to use a human COQ2 structural model in order to predict potential binding sites for its substrates, para-hydroxybenzoate (PHB) and decaprenyl diphosphate (DPP), in a homology-based manner, searching for significant homologs in the Protein Data Bank (PDB), which contain ligands that are structurally similar to these molecules. Further, we determined the location of the human missense single nucleotide mutations, leading to one amino acid substitution affecting COQ2 activity and generating mitochondrial disease, and their effect on COQ2’s structure.

## 2. Materials and Methods

### 2.1. HHpred Search

A human COQ2 (Q96H96) amino acid sequence was downloaded from the UniProt database [21] and used as input for the homology search, performed by HHpred, which is available at the MPI Bioinformatics Toolkit website [22,23], with default parameters. PDB_mmCIF70 was used as a database to select homologs with experimentally solved structures. In contrast to other popular homolog detection tools such as BLAST or PSI-BLAST, HHpred provides a higher sensitivity so that more distant homology relationships can be traced, aligning the query protein with homologs, creating a profile of the hidden Markov model (HMM) from the resulting multiple sequence alignment and comparing it to the profiles of HMMs from the target database [24].

### 2.2. File Parsing

In-house Python 3.8.5 scripts were used and edited in Spyder IDE 5.4.0. First, the PDB Chemical Component Dictionary, featuring all ligands included in PDB structures and detailed information about these molecules, was downloaded as a plain text file (available at https://www.wwpdb.org/data/ccd) and parsed using in-house scripts. For each ligand, the name and the structure, represented as a Simplified Molecular-Input Line-Entry System (SMILES) string, was extracted. The HHpred output was also downloaded as a text file and processed: hits with a probability score higher than 50% and containing ligands were selected.

### 2.3. Molecular Similarity

The similarity between ligands and molecules’ PHB and undecaprenyl phosphate (UP) was determined using a Python script based on a tutorial that is available at the TeachOpenCADD platform [25]. UP was used instead of the actual substrate, DPP, because the latter is not present in the PDB Chemical Component Dictionary. The 3-letter codes identifying PHB and UP in the dictionary were PHB and 5TR, respectively. To determine the degree of similarity between a ligand and query molecule, we obtained their Molecular ACCess System (MACCS) fingerprint, which shows the predefined chemical features that are present. Then, the fingerprints were compared, and the molecular similarity was computed in terms of the Tanimoto coefficient, with values ranging from 0 (lowest similarity) to 1 (highest similarity) [25,26]. Ligands with a Tanimoto coefficient above 0.3 were selected for further studies.

### 2.4. Multiple Sequence Alignment (MSA)

A BLAST 2.15.0 (Basic Local Alignment Search Tool) [27] search with human COQ2 as the query and UniProtKB/SwissProt as the database was performed to retrieve homologs from different organisms. Other homologous sequences were obtained from the UniProtKB database [21]. All sequences were aligned in SeaView 5.0.5 [28], using MUSCLE (MUltiple Sequence Comparison by Log- Expectation) [29]. The MSA was manually edited in Jalview 2.11.2.7 [30].

### 2.5. Two-Dimensional Structure Prediction

COQ2’s secondary structure, disordered regions and transmembrane helices were predicted using PSIPRED 4.0 [31], DISOPRED3 [32] and MEMSAT-SVM (Nugent and Jones 2009), respectively, all of which are available at the PSIPRED Workbench [33].

### 2.6. AlphaFold2 Prediction Model

The structure prediction model for COQ2 was retrieved from the AlphaFold Protein Structure Database [20] and subsequently visualized and customized in PyMOL (The PyMOL Molecular Graphics System, Version 2.0 Schrödinger, LLC, New York, NY, USA.). The final model was exported in PDB format.

### 2.7. Ligand Binding Site Prediction

Putative binding sites were predicted using the Protein Binding Sites (ProBiS) server (http://probis.cmm.ki.si, accessed on 1 September 2023) [34] using the COQ2 model as the query protein, with default settings. The query protein was structurally compared to ligand-bound structures that are available in the PDB. The algorithm then searched for local similarities between them, so that structures with similar regions were selected and aligned to the query protein. Finally, a score that represents structural conservation for each residue in the query protein was computed.

### 2.8. Variant Structure Prediction

Structural modifications induced by mutations were predicted by replacing the mutated residue in the human COQ2 amino acid sequence, which was then introduced into UCSF ChimeraX 1.5 [35]. The predictions were generated using ColabFold [36], run on Google Colab.

### 2.9. Structure Superposition

Homologs with potentially interesting ligands were superposed to the AlphaFold prediction model of COQ2 using the PyMOL “super” command, while the predicted structures containing mutations were superposed to the COQ2 model with the PyMOL “align” command. The first one is appropriate for proteins that share 30% or less sequence identity, whereas the second works for sequences with more than 30% shared identity [37,38].

## 3. Results

### 3.1. Homology Search

The homology search, conducted using HHpred with human COQ2 (Q96H96) as the query, revealed 22 hits with available structures in the PDB, some of them referring to the same homolog, which aligned with more than one region. The hit with the highest score was 4OD4_A, a homolog of UbiA in the archaea *Aeropyrum pernix* K1 (ApUbiA), but this homolog does not contain a ligand. Six other structures had at least one hit with a probability score higher than 50%. Five of them contained ligands: 6M31_B, 8DJM_B, 4TQ3_B, 7Q21_f and 7E1V_H. Results were manually curated to check for contact with the correct PDB chain. Structures without ligands being bound to the correct chain, or ligands associated with different chains, were disregarded (e.g., 7E1V_H and 7Q21_f). In addition, even though the hit with the highest score (4OD4_A) did not contain any ligands, a structure of the same protein with different ligands can be found on the PDB with identifier 4OD5_A [39] and was included in our analysis.

Five structures containing ligands were selected for further analysis: 4OD5_A, the structure of ApUbiA; 6M31_B, a digeranylgeranylglyceryl phosphate synthase from the archaea *Methanocaldococcus jannaschii* DSM 2661 (MjDGGGPase); 8DJM_B, UbiA prenyltransferase domain-containing protein 1, another member of the UbiA family, from the Chinese hamster, *Cricetulus griseus* (CgUBIAD1); 4TQ3_B, a homolog of UbiA from the archaea *Archaeoglobus fulgidus* DSM 4304 (AfUbiA); and 7Q21_f, cytochrome c oxidase polypeptide 4, a component of the respiratory supercomplex in the Gram-positive bacteria *Corynebacterium glutamicum* ATCC 13032. From these, 4OD5_A, 6M31_B and 4TQ3_B were solved by X-ray diffraction (resolutions of 3.56, 2.30 and 2.41 Å, respectively), while the method used for 8DJM_B and 7Q21_f was electron microscopy (resolutions of 3.23 and 2.90 Å, respectively). The list of ligands associated with these structures can be found in Table 1.

### 3.2. Ligand Similarity

**Figure 1:**
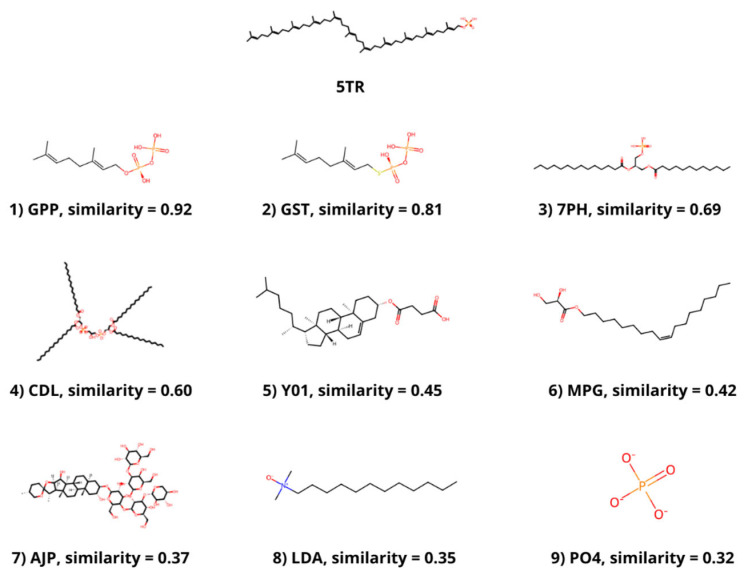
Ligands with a Tanimoto coefficient above 0.3 compared to 5TR. The chemical structure, the component identifier in the PDB Component Dictionary and the value of the Tanimoto coefficient for each ligand are displayed.

The molecular similarity between the ligands that are bound to the selected structures and PHB or a similar molecule bound to DPP, 5TR, was determined. PHB is present in 4OD5_A, but no similar ligand was found in any other selected structure. The most similar molecule to 5TR was geranyl diphosphate (GPP), which was bound to 4TQ3_B, with a Tanimoto coefficient of 0.92. The molecule with the second highest Tanimoto coefficient (0.81) was geranyl S-thiolodiphosphate (GST), which was present in 4OD5_A. Other ligands with a coefficient value higher than 0.3 were phosphatidic acid (7PH) and cardiolipin (CDL) from 7Q21_f; cholesterol hemisuccinate (Y01) and digitonin (AJP) from 8DJM_B; and [(Z)-octadec-9-enyl] (2R)-2,3-bis(oxidanyl)propanoate (MPG), lauryl dimethylamine-n-oxide (LDA) and phosphate ion (PO4) from 6M31_B (Figure 1).

### 3.3. Residues Potentially Involved in Substrate Binding

**Figure 2:**
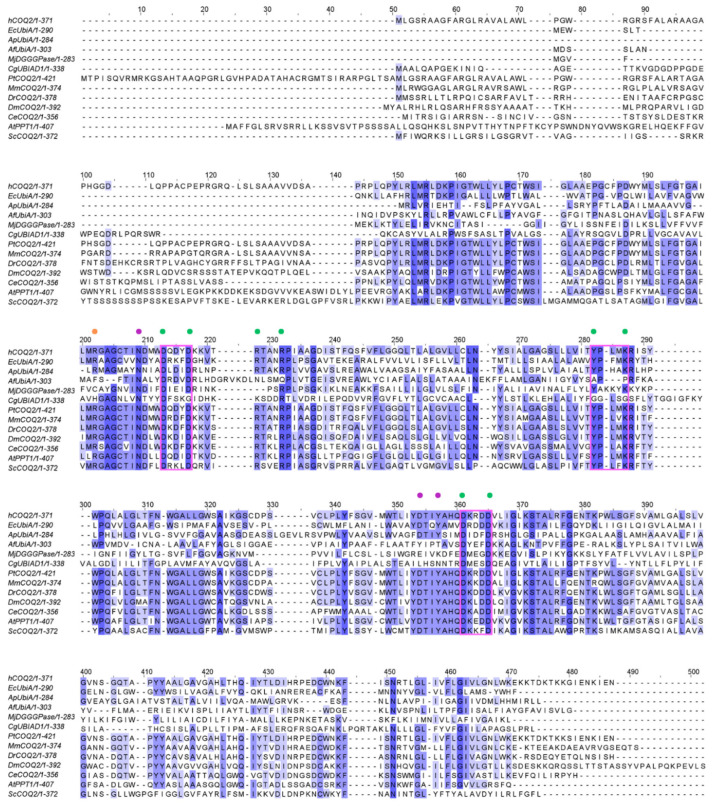
Multiple sequence alignment of human COQ2 (hCOQ2) and some homologs from different organisms, including UbiA from *E. coli* (EcUbiA), UbiA homolog from *A. pernix* (ApUbiA), UbiA homolog from *A. fulgidus* (AfUbiA), DGGGPase from *M. jannaschii* (MjDGGGPase), UBIAD1 homolog from *C. griseus* (CgUBIAD1), COQ2 from *Pan troglodytes* (PtCOQ2), COQ2 from *Mus musculus* (MmCOQ2), COQ2 from *Danio rerio* (DrCOQ2), COQ2 from *Drosophila melanogaster* (DmCOQ2), COQ2 from *Caenorhabditis elegans* (CeCOQ2), PPT1 from *Arabidopsis thaliana* (AtPPT1) and COQ2 from *Saccharomyces cerevisiae* (ScCOQ2). The degree of sequence conservation is indicated in shades of blue: dark (>80%), medium (>60%) and light (>40%). Conserved motifs are represented in pink boxes. Residues located in the proximity of the PPi group and the C1 atom of the tail precursor are denoted by green and purple dots, respectively. The residue associated with PHB binding is depicted with an orange dot.

ApUbiA (4OD5_A) was used as a template to identify residues that are potentially involved in substrate binding in human COQ2 (hCOQ2). Several residues, all of them conserved in hCOQ2 (Figure 2), were proposed to be important for protein–substrate interactions in the ApUbiA structure. When the corresponding residues in EcUbiA were mutated, the activity stopped or was or reduced [39].

Based on structural proximity, the ApUbiA residues Asp54, Asp58, Arg63, Arg67, Tyr115, Lys119, Asp182 and Asp186 may interact with the pyrophosphate (PPi) group of the tail precursor and with two Mg^2+^ ions that are required as cofactors by the UbiA family prenyltransferases. The equivalent residues in hCOQ2 are Asp134, Asp138, Arg143, Arg147, Tyr195, Lys199, Asp255 and Asp259. Additionally, the residues Asn50, Asp175 and Tyr178 (Asn130, Asp248 and Tyr251 in hCOQ2) are located in the proximity of the C1 atom of the tail precursor, and Arg43 is located close to the ligand PHB’s carboxyl group in the ApUbiA structure, which corresponds to Arg123 in hCOQ2 [39].

### 3.4. COQ2 Structural Model

**Figure 3:**
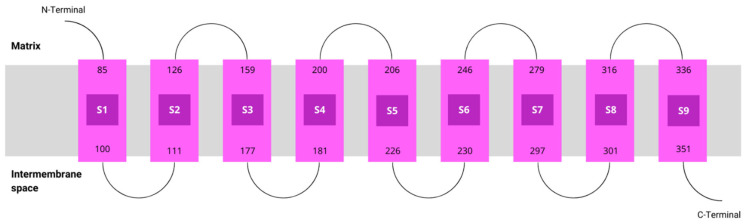
Topology of COQ2. Transmembrane helices predicted by MEMSAT are represented by pink boxes.

According to MEMSAT, hCOQ2 is predicted to contain nine transmembrane regions, called S1–S9. The N-terminal (which bears the signal peptide) and the loops connecting S2–S3, S4–S5, S6–S7 and S8–S9 face the matrix side of the inner mitochondrial membrane, whereas the C-terminal and the loops between S1 and S2, S3 and S4, S5 and S6, and S7 and S8 lie on the intermembrane space (Figure 3).

**Figure 4:**
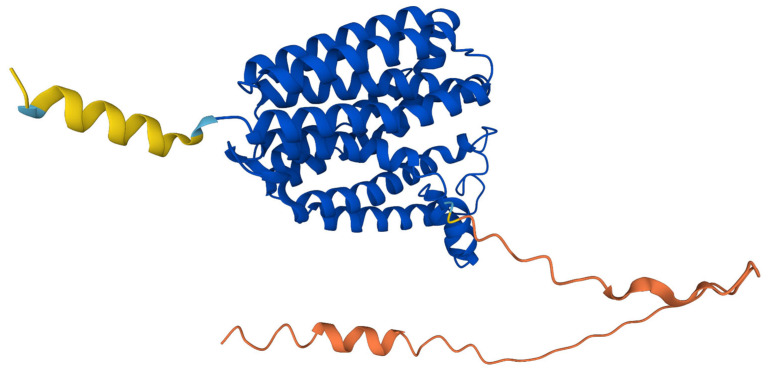
AlphaFold prediction model for COQ2. The colors represent the per-residue confidence score for the prediction: dark blue (very high, pLDDT > 90), light blue (confident, 90 > pLDDT > 70), yellow (low, 70 > pLDDT > 50) and orange (very low, pLDDT < 50). Figure taken from the AlphaFold Protein Structure Database [20].

**Figure 5:**
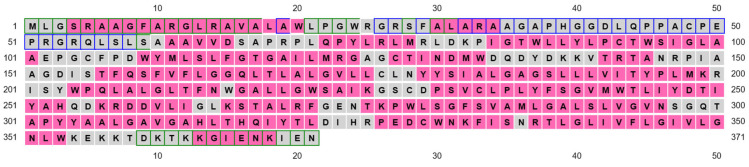
Secondary structure and disordered region prediction for COQ2. Helices predicted by PSIPRED are indicated in pink. Putative disordered regions according to DISOPRED are depicted by blue or green boxes.

**Figure 6:**
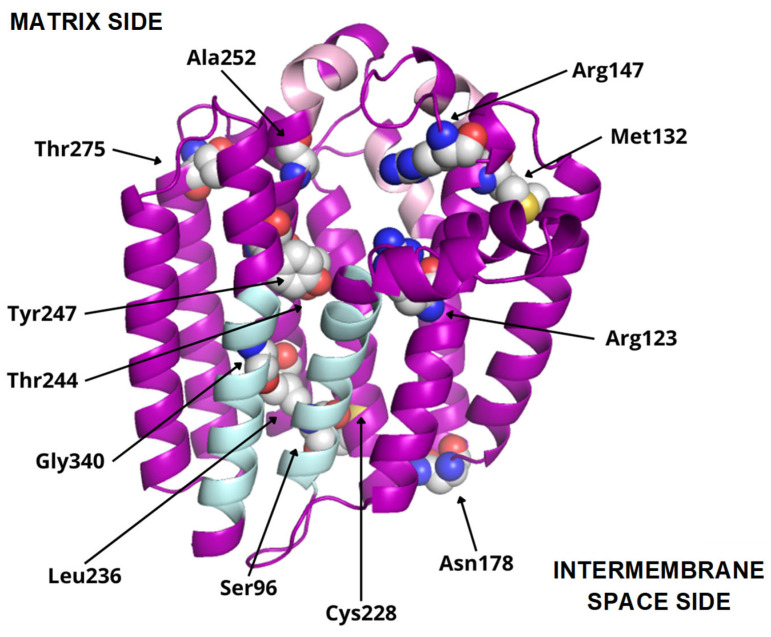
COQ2 structural model (positions 60–354). Residues mutated in primary CoQ deficiency patients are represented as spheres and labeled in black. Conserved motifs are colored in pink. The transmembrane helices S1 and S9, delimiting the putative lateral portal, are indicated in sky blue.

The predicted structure of hCOQ2 (Q96H96) is available in the AlphaFold Protein Structure Database (Figure 4) [20]. This model shows that hCOQ2 is an all-helical protein, which is in line with the results of the secondary structure prediction by PSIPRED (Figure 5). It is composed of several α-helices that are connected by short loops and arranged in a channel-like structure, containing what appears to be a central cavity. The confidence level, measured as a per-residue confidence score (pLDDT), is generally very high (pLDDT > 90, dark blue), except for the N- and C-terminus, which were predicted to be disordered by DISOPRED (Figure 5). The first one has a very low confidence (pLDDT < 50, orange) in positions 1–57, whereas the second mostly shows a low confidence (70 > pLDDT > 50, yellow) from residue 356 to the end of the protein, which is in agreement with the disorder prediction related to the pLDDT score [40,41]. Regions with a pLDDT value lower than 90 were removed in this analysis for clarity, so the final model used in the subsequent analysis is composed of residues 60–354 (Figure 6).

To date, twelve-point mutations in hCOQ2 have been associated with primary CoQ deficiency (Table 2), and most of them are pathogenic according to the prediction performed by Alcázar-Fabra et al. (2021) [1] using SIFT (Sort Intolerant From Tolerant) [42]. Mutated residues are represented as spheres in the model (Figure 6). Based on these, five residues are in predicted transmembrane regions, mostly facing the putative inner surface of the channel. The rest of them can be found on loop regions, five on the matrix side and two facing the intermembrane space.

Additionally, interesting regions were highlighted in the COQ2 model (Figure 6): two conserved Asp-rich motifs ranging from positions 134 to 138 (Asp134XXXAsp138) and 255 to 259 (Asp255XXXAsp259), an additional motif in positions 195–199 (Tyr195XXXLys199) and a lateral opening delimited by S1 and S9. The Asp-rich motifs are a signature of the UbiA family members, which could be important for activity or substrate interaction, located in the loop regions connecting S2–S3 and S6–S7, whereas the third motif is found on the loop between S4 and S5, all of them on the matrix side. The lateral opening has been described in some UbiA homologs such as ApUbiA, AfUbiA and MjDGGGPase [17,39,46,47].

### 3.5. Structural Superposition of Homologous Structures Relative to COQ2

When superposed to the hCOQ2 model, the most similar structure, i.e., the one with the lower root-mean square deviation (RMSD), was 4TQ3_B (2.901 Å), followed by 4OD5_A (3.477 Å), 6M31_B (4.516 Å), 8DJM_B (4.992 Å) and, lastly, 7Q21_f (18.917 Å). Given its high RMSD value, 7Q21_f was excluded from further analysis.

**Figure 7:**
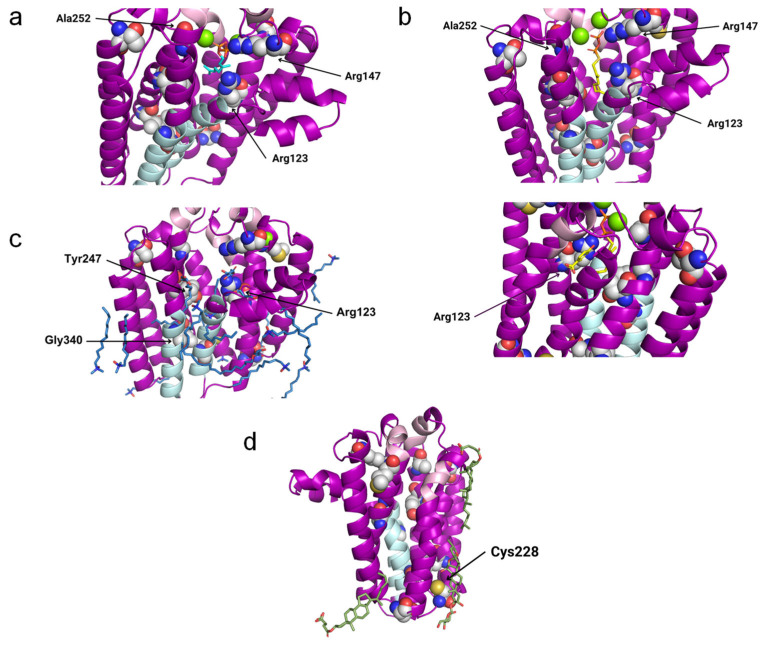
Potentially interesting ligands modeled onto the COQ2 structural model. The conserved motifs and the lateral portal are depicted in pink and sky blue, respectively, while residues associated with primary CoQ deficiency are shown as spheres. The mutated residues that lie close to a potentially interesting ligand are labeled in black. The superposed structures of the homologous proteins are removed for clarity. (**a**) GPP in cyan and two Mg^2+^ ions in green from 4TQ3_B. (**b**) GST and pHB in yellow and two Mg^2+^ ions in green from 4OD5_A. (**c**) MPG and LDA in blue and a Mg^2+^ ion in green from 6M31_B. (**d**) Y01 and AJP in green from 8DJM_B.

In the 4TQ3_B structure (AfUbiA), the GPP is located inside the central cavity, with the PPi group located at the matrix side. The above-indicated conserved motifs and two Mg^2+^ ions are in the proximity of the PPi group. Three residues that are mutated in CoQ primary deficiency patients are close to GPP: Arg123, Arg147 and Ala252, all of which are predicted to be pathogenic (Figure 7a).

In 4OD5_A (ApUbiA), both the GST and the PHB can be found inside the central cavity. The PPi group from GST is facing the matrix, with two Mg^2+^ ions, the Asp-rich motifs, the Tyr195XXXLys199 motif and residue Arg147 in its proximity, whereas pHB is close to Arg123. Ala252 is close to one of the Mg^2+^ ions (Figure 7b). This suggests that the Mg^2+^ ions play an important role in the binding of the reactive end of GST to pHB.

In 6M31_B (MjDGGGPase), several MPG and LDA molecules are located inside the cavity, in the lateral portal and around the protein. In the central cavity, three mutated residues (Arg123, Tyr247 and Gly340) are close to the ligands. A Mg^2+^ ion is bound to the structure on the matrix side and near the mutated residue Arg123, but in a more peripheral position compared to 4TQ3_B and 4OD5_A (Figure 7c).

In 8DJM_B (CgUBIAD1), the Y01 and AJP are situated around the protein. AJP is found in the proximity of Cys228, a residue that is mutated in primary CoQ deficiency patients (Figure 7d).

All these results generally agree with the predictions made by ProBiS [34]. The structure with the highest confidence is 4OD5, closely followed by 4TQ3, among others. The ligands bound to these structures can be found inside the central cavity of hCOQ2, close to the matrix side, supporting our findings. Furthermore, some of the binding sites that were predicted by ProBiS are consistent with the residues putatively involved in substrate binding that were discussed previously: Asn130, Arg143, Arg147, Tyr195, Lys199 and Tyr251.

### 3.6. Effects of Mutations in COQ2 Structure

**Figure 8:**
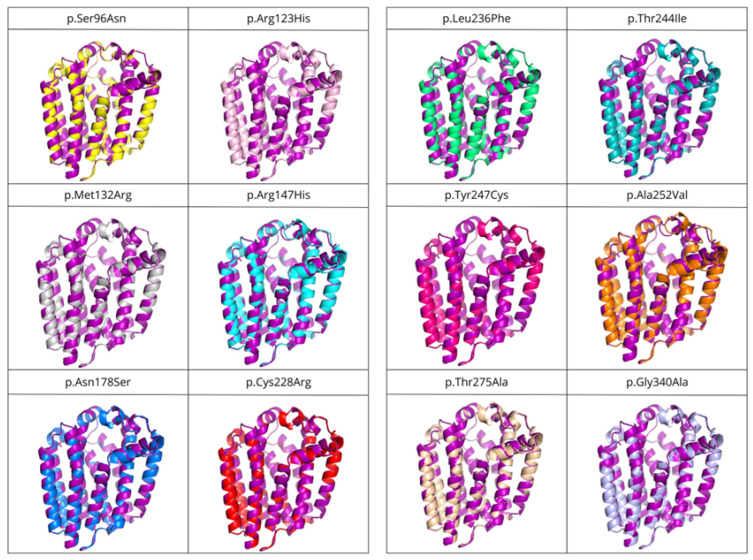
Structural superposition of variants associated with primary CoQ deficiency onto the COQ2 model. Each structure is represented in a different color: p.Ser96Asn (yellow), p.Arg123His (pink), p.Met132Arg (grey), p.Arg147His (cyan), p.Asn178Ser (blue), p.Cys228Arg (red), p.Leu236Phe (green), p.Thr244Ile (teal), p.Tyr247Cys (magenta), p.Ala252Val (orange), p.Thr275Ala (wheat), p.Gly340Ala (light blue) and wild-type (purple).

The structures of the twelve variants with mutations associated with primary CoQ deficiency were predicted and then superposed to the COQ2 model. A list of the obtained variants and the RMSD values, in order from lowest to highest (i.e., from more similar to the wild-type to more divergent), can be found in Table 3. There does not seem to be a correlation between the pathogenicity of the mutation and the structural similarity of the variants to the wild-type. As expected, point mutations do not produce great global impact on the whole structure of the protein, with RMSD values below 0.3 for all the variants. However, locally, some of them cause small alterations in the loop between S6 and S7 on the matrix side, as well as the N-terminal region, even when the modification takes place elsewhere in the protein (Figure 8). These small modifications could be related to the loss of interaction with the rest of the members of the CoQ-synthome, explaining their high pathogenic effect.

## 4. Discussion

The tertiary structure prediction from amino acidic sequences has always been a major challenge in bioinformatics, but recent advances in artificial intelligence have brought about a revolution in this field [48]. AlphaFold2 is a machine learning method that not only predicts protein structures with high accuracy using both evolutionary and geometric information, but also determines a confidence value on a residue level. This is possible thanks to an innovative architecture based on artificial neural networks [40,41]. This technology was applied to all known sequences, and the AlphaFold Protein Structure Database currently holds more than 200 million predicted structures [20], including hCOQ2.

Our goal was to leverage the homologous relationship of hCOQ2 to other members of the UbiA family with ligands containing experimentally solved structures, including UbiA homologs from the thermophilic archaea *A. pernix* (ApUbiA) and *A. fulgidus* (AfUbiA); a DGGGPase enzyme from another archaeum, *M. jannaschii* (MjDGGGPase); and a UBIAD1 homolog from the Chinese hamster, *C. griseus* (CgUBIAD1). Proteins from the UbiA family are integral prenyltransferases, responsible for the synthesis of a wide range of molecules: CoQ and other quinones such as menaquinone and plastoquinone, vitamin E, chlorophyll, heme, secondary metabolites, components of the cell wall in mycobacteria and membrane lipids in archaea [17,49,50].

It has been predicted that hCOQ2 contains nine transmembrane regions connected by loops, with the N-terminus facing the matrix side and the C-terminus facing the intermembrane space [51]. This topology was previously reported by Desbats et al. (2016), except that a different isoform of the protein was used in their analysis [51]. The hCOQ2 gene has four possible start codons and the isoform obtained from the transcript that was generated from the most downstream ATG (called ATG4), which was used in the present study, is the most common one, with 371 amino acids [1,51]. Moreover, the AlphaFold model for hCOQ2 shows with a high confidence that it is an all-helical protein organized in a channel-like structure with a central cavity, which is consistent with the PDB structures of the homologous proteins ApUbiA, AfUbiA, MjDGGGPase and CgUBIAD1: 4OD5_A, 4TQ3_B, 6M31_A and 8DJM_B, respectively [17,39,46,47]. When the AlphaFold model is superposed to these structures, the RMSD values are generally globally good, despite the low similarity in their sequences. In contrast, the highest RMSD value by far is obtained, as expected, with 7Q21_f, a structure of the cytochrome c oxidase subunit from *C. glutamicum*, because it is the only selected homolog that does not belong to the UbiA family.

It is noteworthy that the superposition of UbiA homologs from archaea to hCOQ2 provides lower RMSD values than the homolog from the only mammal, the Chinese hamster. In this case, the technique that is used for structure determination must be taken into consideration. The structure of the archaeal proteins was determined by X-ray crystallography, which usually yields a higher resolution than electron microscopy, which was used for 8DJM_B. The exception is 4OD5_A, solved by X-ray diffraction, but with the worst resolution among all the selected structures. This could explain why 4TQ3_B (AfUbiA) provides the lowest RMSD value instead of 4OD5_A (ApUbiA), even when the second one is closer, in sequence, to hCOQ2 [49]. It is also interesting that, according to previous phylogenetic and clustering studies, the UbiA/COQ2 cluster is more distant from MenA/UBIAD1 than from AfUbiA or MjDGGGPase [17,49], suggesting an earlier divergence of UbiA/COQ2 and MenA/UBIAD1.

Another important observation is that the HHpred output includes 4OD4_A, the apo-state structure of ApUbiA, but not 4OD5_A, the same structure but bound to ligands, because it only takes into consideration the most similar structure for a given protein, the representative, to avoid redundancy. In future approaches, the relationship between the structure and the nature of the ligands would help to discover the binding motives of another representative of the CoQ-synthome. Another problem is that a small number of significant homologs with solved structures were identified and, therefore, the information obtained from them is limited, which is not surprising given that transmembrane structure determination is a challenging matter [52].

The members of the UbiA family catalyze the transfer of hydrophobic chains derived from isoprene or phytol to other compounds that act as acceptors, generally aromatic molecules, providing hydrophobicity [53]. hCOQ2 is responsible for the addition of an isoprenoid chain (DPP in humans) to PHB, which is the precursor of CoQ’s redox-active head. The search for similar ligands bound to the selected structures yielded nine molecules with a Tanimoto coefficient of at least 0.3 compared to 5TR, a molecule resembling the substrate DPP. The most similar ligands are GPP, a precursor of DPP that can be used in vitro by EcUbiA [39,54], and GST, a non-metabolizable analogue of GPP [39], followed by other molecules with long linear chains and an artificial derivative of cholesterol, among others.

When the structures 4TQ3_B (AfUbiA) and 4OD5_A (ApUbiA), containing GPP and GST, respectively, are superposed to hCOQ2, these ligands are found inside the central cavity, with the PPi group positioned towards the matrix side and two Mg^2+^ ions nearby, in the proximity of the motifs located on the matrix loops and two residues that are mutated in primary CoQ deficiency patients (Arg147 and Ala252). It has been proposed that the motifs found in the matrix loops play an important role in substrate binding and/or activity, probably involving the coordination of the Mg^2+^ ions that bridge the CoQ tail precursor with the Asp residues in the motifs and are necessary for the reaction with PHB [17,39,46,47]. We speculate that these residues are essential for the stability of the interaction with the isoprenoid tail, explaining why these mutations are incompatible with life [55].

On the contrary, mutations affecting the residues Arg147 and Ala252 [51], located on the matrix side of hCOQ2, are found in CoQ-deficient patients [1]. The equivalent residue of Arg147 in ApUbiA is predicted to be involved in recognition of the PPi group in the polyisoprenoid chain, and mutating its counterpart in EcUbiA causes a drastic reduction in the enzymatic activity. Meanwhile, Ala252 is next to the residue Tyr251, whose counterpart in ApUbiA is thought to be close to the C1 atom of the isoprenoid chain [39]. Furthermore, the pathogenic variants p.Arg147His and p.Ala252Val were previously proposed to disrupt the interaction with the PPi group and one of the Mg^2+^ ions, respectively [49]. p.Arg147His was found in heterozygosis together with another mutation, p.Asn178Ser, in a 2-year-old boy who suffered steroid-resistant nephrotic syndrome (SRNS) [56], while p.Ala252Val was present in twins with a wide range of symptoms who died a few months after their births [57].

6M31_B provides information related to ligands around the lateral portal that is defined by the transmembrane regions S1 and S9. This opening may be responsible for product release into the inner mitochondrial membrane in eukaryotes or the plasma membrane in prokaryotes [17,39,46]. If hCOQ2 really acts as an anchor site for the CoQ-synthome in humans, it is possible that the product of this protein is not released immediately to the inner mitochondrial membrane and instead stays inside the cavity as the redox head, which is further modified by the other COQ polypeptides until the last product, CoQ_10_, is finally released through the lateral portal. The lateral portal has been observed in ApUbiA, AfUbiA and MjDGGGPase, in accordance with the idea proposed by Chen et al. (2022) that binding of the substrate induces the opening of the portal [46].

Conversely, none of the ligands bound to the selected structures were similar to the aromatic substrate, PHB, which could indicate that proteins of the UbiA family display a higher specificity for linear substrates. It is also worth noting that some alternative aromatic substrates have been suggested in different organisms, including para-aminobenzoic acid in yeast and phenolic compounds such as kaempferol, resveratrol and p-coumaric acid in mammals [12]. Taking into consideration that different substrates of the aromatic head can successfully bind to the isoprene tail, the specificity for the aromatic substrate seems to be less strong than for the isoprene tail. This allows for different head substrates to have been considered in the treatment of CoQ_10_ deficiency due to a dysfunction of the head-modifying CoQ-synthome members [1,58]. Further, this interaction must occur in the matrix side of hCOQ2, permitting the interaction of this part of the molecule with the head-modifying enzymes in the CoQ-synthome. This is consistent with the hypothesis that hCOQ2 functions as an anchor site for the CoQ-synthome and with docking studies published by Herebian et al. (2017) [55], but it does not agree with the putative binding site of this ligand in 4OD5_A (ApUbiA) [39], a residue located in a central position inside the cavity that is equivalent to one of the amino acids that are mutated in primary CoQ deficiency patients, Arg123 [1]. Mutating the counterpart of this residue in EcUbiA has a negative impact on both the enzymatic activity and the interaction with PHB. However, our results reveal that the lateral chain of Arg123 clashes with PHB when 4OD5_A is superposed to the AlphaFold model of hCOQ2, which is in line with the ideas of Huang et al. (2014), who concluded that the structure of AfUbiA does not support this binding site for the aromatic substrate, because it lies too close to the polyprenyl chain [47]. There are also ligands in the proximity of Arg123 in structure 6M31_B. This residue, therefore, could instead interact with the linear substrate, which may explain its importance, whereas the pHB binding site could lie elsewhere. For instance, Herebian et al. (2017) have proposed alternative binding sites for this ligand in different positions of the central cavity [55]. Alternatively, it could simply mean that there are differences between members of the UbiA family concerning the binding site of the aromatic substrate, which explains the different aromatic substrates that can be used by hCOQ2.

Interestingly, the different mutations studied did not produce great changes in the structure of the protein. Some of them produced small alterations in the loop between S6 and S7 and the N-terminal region, both on the matrix side. Taking into consideration their pathogenic effect due to the disruption of the activity of hCOQ2, these small modifications can be associated with a loss of interaction with the rest of the members of the CoQ-synthome. Further experiments in this direction must be performed since, to date, the whole structure of the CoQ-synthome has been elusive.

## 5. Conclusions

Taken together, our results help improve the understanding of hCOQ2, providing an updated schematic representation of its topology, identifying possible substrate binding sites and interpreting some of the pathogenic mutations related to primary CoQ deficiency. It is important, nevertheless, to note that they are mainly based on predictions. AlphaFold, albeit highly useful, also has limitations. For instance, it is not able to predict ligands [59], explaining why it was combined with a homology-based approach here, with all the associated limitations. Thus, it would be of interest to solve the structure of hCOQ2 and other members of the UbiA family both in the apo- and substrate-bound state, as well as to study possible protein–protein interactions involving hCOQ2, especially with other COQ proteins that are thought to be part of the CoQ-synthome, with the purpose of demonstrating the anchor site hypothesis.

## Figures and Tables

**Table 1:** List of significant HHpred hits with ligands. The PDB identifier and a description of the protein are displayed. For each ligand, the name and identifier in the PDB Chemical Component Dictionary are included.

PDB ID	Protein	Ligands
4OD5_A	UbiA homolog from *Aeropyrum pernix* K1	p-hydroxybenzoic acid (PHB)
		Geranyl S-thiolodiphosphate (GST)
		Magnesium ion (MG)
6M31_B	Digeranylgeranylglyceryl phosphate synthase from *Methanocaldococcus jannaschii* DSM 2661	[(Z)-octadec-9-enyl] (2R)-2,3-bis(oxidanyl)propanoate (MPG)
		Lauryl dimethylamine-n-oxide (LDA)
		Phosphate ion (PO4)
		Magnesium ion (MG)
8DJM_B	UbiA prenyltransferase domain-containing protein from *Cricetulus griseus*	Cholesterol hemisuccinate (Y01)
		Digitonin (AJP)
4TQ3_B	UbiA homolog from *Archaeoglobus fulgidus* DSM 4304	Geranyl diphosphate (GPP)
		Magnesium ion (MG)
7Q21_f	Cytochrome c oxidase polypeptide 4 from *Corynebacterium glutamicum* ATCC 13032	Phosphatidic acid (7PH)
		Cardiolipin (CDL)
		Tridecane (TRD)

**Table 2:** List of mutations associated with primary CoQ deficiency and their location in the protein. The pathogenicity status of the variants predicted by SIFT [1,42] and reported in the databases ClinVar (https://www.ncbi.nlm.nih.gov/clinvar/) [43], Leiden Open Variation Database (LODT) (https://www.lovd.nl/3.0/home) [44] and Franklin (https://franklin.genoox.com/clinical-db/home) [45] is indicated.

Amino Acid Modification	Location	SIFT	ClinVar	LOVD	Franklin
Ser96Asn	Transmembrane helix S1	Pathogenic	Pathogenic	Not classified	Likely pathogenic
Arg123His	Transmembrane helix S2	Pathogenic	Uncertain significance	Not classified	Uncertain significance
Met132Arg	Loop between S2 and S3 (matrix side)	Pathogenic	Not provided	Affects function	Likely pathogenic
Arg147His	Loop between S2 and S3 (matrix side)	Pathogenic	Pathogenic/ Likely pathogenic	Probably affects function	Pathogenic
Asn178Ser	Loop between S3 and S4 (intermembrane space side)	Tolerated	Conflicting classifications of pathogenicity	Probably affects function/ Affects function	Likely pathogenic
Cys228Arg	Loop between S5 and S6 (intermembrane space side)	Pathogenic	Not reported	Not reported	Uncertain significance
Leu236Phe	Transmembrane helix S6	Pathogenic	Uncertain significance	Not reported	Uncertain significance
Thr244Ile	Transmembrane helix S6	Tolerated	Uncertain significance	Not classified	Uncertain significance
Tyr247Cys	Loop between S6 and S7 (matrix side)	Pathogenic	Likely pathogenic	Affects function	Likely pathogenic
Ala252Val	Loop between S6 and S7 (matrix side)	Pathogenic	Not provided	Effect unknown	Uncertain significance
Thr275Ala	Loop between S6 and S7 (matrix side)	Tolerated	Not reported	Not reported	Uncertain significance
Gly340Ala	Transmembrane helix S9	Pathogenic	Uncertain significance	Not reported	Uncertain significance

**Table 3:** List of RMSD values obtained by the superposition of the structures containing pathogenic or tolerated mutations related to primary CoQ deficiency onto the COQ2 model.

Variant	SIFT Prediction	RMSD (Å)
p.Ser96Asn	Pathogenic	0.183
p.Met132Arg	Pathogenic	0.199
p.Leu236Phe	Pathogenic	0.207
p.Arg147His	Pathogenic	0.210
p.Asn178Ser	Tolerated	0.225
p.Gly340Ala	Pathogenic	0.237
p.Tyr247Cys	Pathogenic	0.246
p.Cys228Arg	Pathogenic	0.247
p.Thr244Ile	Tolerated	0.247
p.Arg123His	Pathogenic	0.256
p.Thr275Ala	Tolerated	0.257
p.Ala252Val	Pathogenic	0.268

## Data Availability

Data is contained within the article.
